# P311 promotes renal fibrosis via TGFβ1/Smad signaling

**DOI:** 10.1038/srep17032

**Published:** 2015-11-30

**Authors:** Zhihui Yao, Sisi Yang, Weifeng He, Lian Li, Rui Xu, Xiaorong Zhang, Haisheng Li, Rixing Zhan, Wei Sun, Jianglin Tan, Junyi Zhou, Gaoxing Luo, Jun Wu

**Affiliations:** 1 Institute of Burn Research, State Key Laboratory of Trauma, Burn and Combined Injury, Southwest Hospital, The Third Military Medical University, Chongqing 400038, China; 2 Chongqing Key Laboratory for Disease Proteomics, Chongqing 400038, China; 3 Department of Nephrology, Southwest Hospital, The Third Military Medical University, Chongqing 400038, China

## Abstract

P311, a gene that was identified in 1993, has been found to have diverse biological functions in processes such as cell proliferation, migration and differentiation. However, its role in fibrosis is unknown. We previously observed that P311 is highly expressed in skin hypertrophic scars. In this study, P311 over-expression was detected in a subset of tubular epithelial cells in clinical biopsy specimens of renal fibrosis; this over-expression, was found concurrent with α-smooth muscle actin (α-SMA) and transforming growth factor beta1 (TGFβ1) expression. Subsequently, these results were verified in a mouse experimental renal fibrosis model induced by unilateral ureteral obstruction. The interstitial deposition of collagen, α-SMA and TGF-β1 expression, and macrophage infiltration were dramatically decreased when P311 was knocked out. Moreover, TGFβ/Smad signaling had a critical effect on the promotion of renal fibrosis by P311. In conclusion, this study demonstrate that P311 plays a key role in renal fibrosis via TGFβ1/Smad signaling, which could be a novel target for the management of renal fibrosis.

P311, also known as PTZ17, is primarily expressed in the mouse embryonic brain and persists at a high level in the cerebellum, hippocampus and olfactory bulb during adulthood^1^. The P311 gene has been mapped to the chromosome 5 in humans and the chromosome 18 in mice, and it encodes an 8-kDa intracellular protein that contains 68 amino acids and does not belong to any known protein family. The protein’s N-terminus contains a PEST domain (rich in Pro, Glu, Ser and Thr), which is highly conserved among humans, mice and chickens. The PEST domain functions in targeting proteins for degradation via the ubiquitin/proteasome pathway and in protein-protein interactions and activation. The PEST domain was originally identified in short-lived proteins, such as transcription factors, cytokines and signaling molecules^2^,^3^. The half-life of P311 protein is approximately 5 minutes; it is rapidly degraded by Met-HGF/SF, the lactacystin-sensitive ubiquitin/proteasome system and an unidentified metalloprotease^1^. P311 functions in nerve and lung regeneration^4^,^5^, glioblastoma invasion^6^,^7^, blood pressure homeostasis^8^, myofibroblast differentiation and amoeboid-like migration^9^,^10^,^11^. P311-deficient mice have no overt abnormalities, but they exhibit altered behavioral responses in learning and memory^12^. Additionally, P311 is involved in the affective, not the sensory, component of pain^13^, which is consistent with P311 expression in the nervous system.

Renal fibrosis is irreversible and progressive in chronic kidney disease (CKD) and is characterized by the accumulation of extracellular matrix (ECM) components in the glomeruli and tubular interstitium^14^. Regardless of the initial cause, renal fibrogenesis is a dynamic and converging process in which almost all kidney cell types participate. The activation of matrix-producing cells is considered a central event in renal fibrogenesis, as it involves fibroblasts, tubular epithelial cells, vascular smooth muscle cells and a subset of macrophages^15^. Tubular epithelial cells are the primary effector cells that contribute to the irreversible progression of late-stage renal fibrosis. Tubular epithelial cells undergo the phenotypic process of epithelial-mesenchymal transition (EMT) in microenvironments containing various pro-fibrotic cytokines, particularly transforming growth factor beta (TGF-β)^16^,^17^. In CKD, TGF-β over-expression induces renal fibrosis, while TGF-β inhibition decreases chronic renal fibrosis^18^,^19^,^20^. TGF-β is a crucial regulator of EMT that mediates many pathological CKD-associated processes^14^,^21^ and causes tubular epithelial cells to begin to express vimentin (a fibroblast marker) and α-smooth muscle actin (α-SMA, a myofibroblast marker)^17^,^22^,^23^.

In our previous studies, we found that P311 was highly over-expressed in early hypertrophic scars and was involved in the pathogenesis of hypertrophic scars^10^,^24^. Our previous findings led us to explore whether P311 plays a role in renal fibrogenesis. Thus, in this study, we investigated the potential roles of P311 in renal fibrosis and further studied the underlying molecular mechanism.

## Results

### P311 expression and distribution in human renal fibrosis

**Figure 1:**
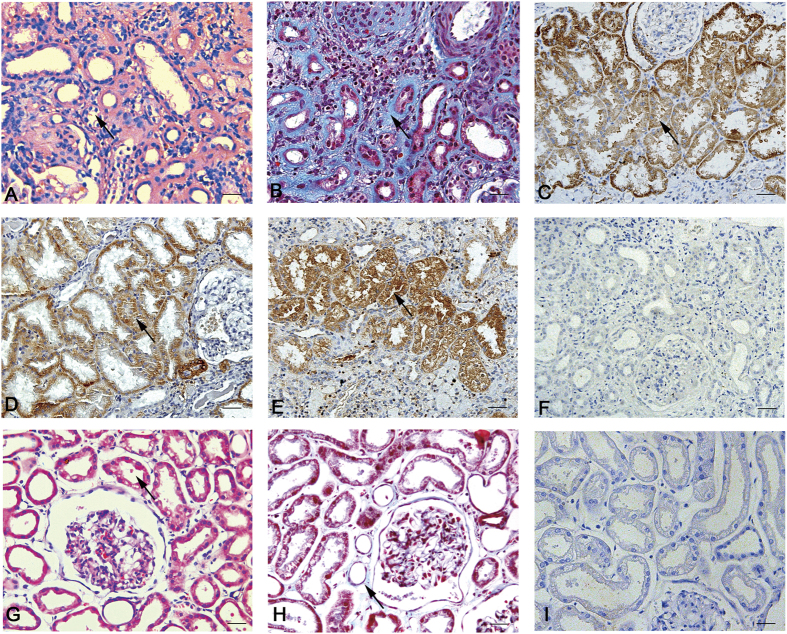
Interstitial collagen deposition and P311, α-SMA and TGFβ1 expression in human renal fibrosis. (**A**) Hematoxylin and eosin staining of human renal fibrosis samples (n = 22). Black arrowhead indicates the remarkable tubular atrophy. (**B**) Masson trichrome staining of human renal fibrosis samples (n = 22). Black arrowhead indicates the interstitial collagen fibril deposition. (**C**) P311 immunoreactivity in the cytoplasm of some tubular epithelial cells from human renal fibrosis samples (n = 22). Black arrowhead indicates the P311-positive region. (**D**) α-SMA immunoreactivity in the cytoplasm of some tubular epithelial cells from human renal fibrosis samples (n = 22). Black arrowhead indicates the α-SMA-positive region. (**E**) TGFβ1 immunoreactivity in the cytoplasm of some tubular epithelial cells from human renal fibrosis samples (n = 22). Black arrowhead indicates the TGFβ1-positive region. (**F**) Negative control for the immunohistochemical staining on human renal fibrosis samples (n = 22). (**G**) Hematoxylin and eosin staining of healthy human kidney samples (n = 2). Black arrowhead indicates the normal renal tubules. (**H**) Masson trichrome staining of healthy human kidney samples (n = 2). Black arrowhead indicates the little interstitial collagen fibril. (**I**) P311 is negative in normal human renal tubular epithelial cells (n = 2). Scale bar: 100μm.

We investigated 22 human clinical biopsy specimens of renal fibrosis and 2 normal human kidney tissue samples, which were diagnosed via histopathology (Table 1). Remarkable tubular dilation and atrophy, interstitial expansion and abundant inflammatory cell infiltration were observed after hematoxylin and eosin (HE) staining (Fig. 1A). Masson trichrome staining revealed a large amount of interstitial collagen fibril deposition (Fig. 1B). P311 protein levels were significantly increased in the cytoplasm of some tubular epithelial cells in human renal fibrosis tissues samples, as evidenced by immunohistochemical analysis (Fig. 1C). In addition, we examined α-SMA and TGF-β1 expression and found that both α-SMA and TGF-β1 were highly expressed in the cytoplasm of some tubular epithelial cells in human renal fibrosis tissues, and their expression pattern was similar to that of P311 (Fig. 1C–F). Normal human kidney tissue samples indicate the normal renal tubules and the little interstitial collagen fibril (Fig. 1G,H). However, tubular epithelial cells in normal human kidney tissues were negative for P311 (Fig. 1I); these cells were also negative for TGF-β1 and α-SMA (data not shown). These results indicated the possible role of P311 in human renal fibrogenesis.

### P311 expression and distribution in the adult mouse kidney after UUO

**Figure 2:**
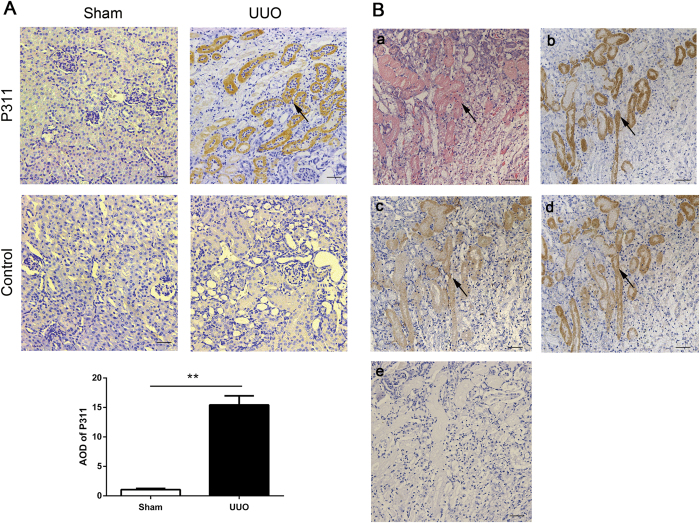
Increased levels and cellular localization of P311 in mouse renal fibrosis. (**A**) P311 expression and distribution in kidney tissues after sham-operation (Sham) or unilateral ureteral obstruction (UUO) in adult mice, as evidenced by immunohistochemistry. Black arrowhead indicates the P311-positive region. P311-positive region was quantified in stained sections from the Sham (n = 6) and UUO (n = 6) groups. Data are presented as the mean ± SD. *P < 0.05; **P < 0.01. (B) TGFβ1 and α-SMA as well as acidophilic degeneration seem to be co-localized with P311 in some tubular epithelial cells within serial sections from mouse obstructed kidneys (n = 6). (**a**) Hematoxylin and eosin staining of mouse renal fibrosis samples (n = 6). Black arrowhead indicates acidophilic degeneration. (**b**) P311 immunoreactivity in the cytoplasm of some tubular epithelial cells from mouse renal fibrosis samples (n = 6). Black arrowhead indicates the P311-positive region. (**c**) α-SMA immunoreactivity in the cytoplasm of some tubular epithelial cells from mouse renal fibrosis samples (n = 6). Black arrowhead indicates the α-SMA-positive region. (**d**) TGFβ1 immunoreactivity in the cytoplasm of some tubular epithelial cells from mouse renal fibrosis samples (n = 6). Black arrowhead indicates the TGFβ1-positive region. (**e**) Negative control for the immunohistochemical staining on mouse renal fibrosis samples (n = 6). (**A**,**B**) are representative of at least three similar experiments. Scale bar: 100μm.

To determine whether the increased P311, TGF-β1 and α-SMA levels in renal fibrosis in humans were also present in mice, we induced renal interstitial fibrosis by unilateral ureteral obstruction (UUO). HE staining revealed marked tubular dilation and atrophy, interstitial matrix deposition and inflammatory cell infiltration 7 days after UUO. Meanwhile, some tubular epithelial cells in obstructed kidneys became swollen and exhibited acidophilic degeneration (data not shown). To examine P311 localization, we performed immunohistochemistry with an antibody against P311. P311 was primarily localized to the cytoplasm of some tubular epithelial cells in P311^+/+^ mice after UUO. However, we did not detect P311 expression in tubular epithelial cells from mice in the sham operation (Sham) group (Fig. 2A, 15.42-fold difference, P < 0.001). We examined TGFβ1 and α-SMA expression in relation to P311 expression. The TGFβ1- and α-SMA-positive regions and the acidophilic degeneration regions seem to be co-localized with the P311-positive regions in some serial sections of tubular epithelial cells (Fig. 2B). Together, these data indicated that P311 expression increased and P311 seem be co-localized with TGFβ1 and α-SMA in obstructed kidneys in mice, which may contribute to the pathology of renal fibrogenesis.

### Renal fibrosis after UUO was dramatically inhibited after knocking out P311

**Figure 3:**
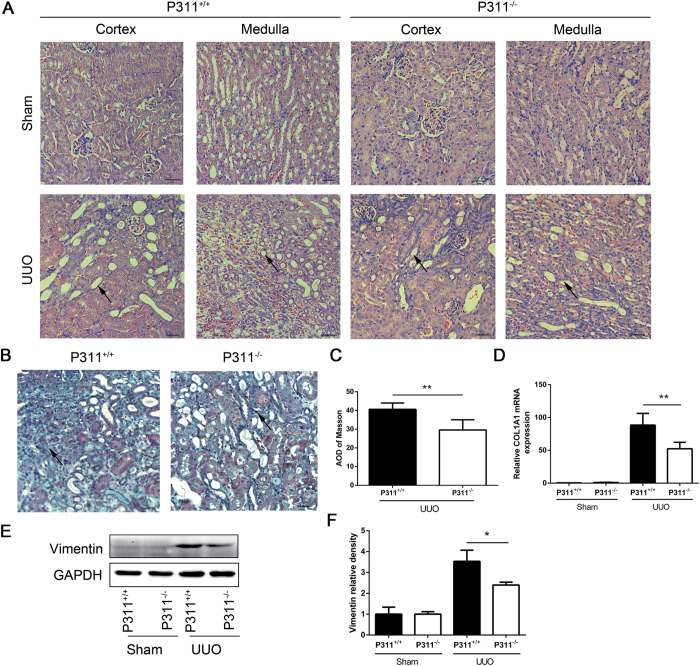
P311 deficiency suppresses renal fibrosis in UUO mice. (**A**) Histologic changes in the cortex and medulla of kidneys from P311^+/+^ and P311^−/−^ mice. Representative hematoxylin and eosin staining of renal cortex and medulla sections from the Sham and UUO groups of both P311^+/+^ (left) (n = 6) and P311^−/−^ (right) (n = 6) mice on day 7. Black arrowhead indicates the remarkable tubular atrophy. (**B**) Representative photomicrographs of Masson’s trichrome staining of kidney sections from P311^+/+^ (n = 6) and P311^−/−^ (n = 6) mice at day 7 after UUO. Black arrowhead indicates the interstitial collagen fibril deposition. (**C**) Fibrotic areas were quantified in stained sections from P311^+/+^ (n = 6) and P311^−/−^ (n = 6) mice at day 7 after UUO. (**D**) RNA was isolated from kidneys of P311^+/+^ (n = 6) and P311^−/−^ (n = 6) mice after UUO or sham operation. Collagen I mRNA expression was determined by real-time PCR. (**E**) Western blot analysis of vimentin protein levels. (**F**) Quantification of vimentin protein levels in each treatment group (n = 3 per group). A-F are representative of at least three similar experiments. Scale bar: 100 μm. Data are presented as the mean ± SD. *P < 0.05; **P < 0.01.

The above findings suggested a potential functional role for P311 in renal fibrosis. To examine this hypothesis, we analyzed P311^−/−^ mice and found that obstructed kidneys in both P311^+/+^ and P311^−/−^ mice exhibited tubular dilation and atrophy, interstitial matrix deposition and inflammatory cell infiltration (Fig. 3A). These changes were more severe in P311^+/+^ mice. Quantitative analysis of Masson trichrome-positive areas revealed an approximately 1.43-fold increase in interstitial collagen deposition in P311^+/+^ mice compared to P311^−/−^ mice after UUO (Fig. 3B,C, P = 0.005). Real-time PCR showed that collagen I mRNA levels were very low in the P311^+/+^ and P311^−/−^ Sham groups, but were significantly increased in the UUO groups. However, collagen I mRNA expression increased more in P311^+/+^ mice than in P311^−/−^ mice after UUO (Fig. 3D, 1.69-fold difference, P = 0.010). Vimentin expression was significantly higher in obstructed kidneys from P311^+/+^ mice compared to P311^−/−^ mice, as determined by western blot (Fig. 3E,F, 1.52-fold difference, P = 0.024). Therefore, these results suggested that P311 promotes renal fibrogenesis in a mouse model of UUO.

### P311 promoted α-SMA expression in a mouse model of UUO

**Figure 4:**
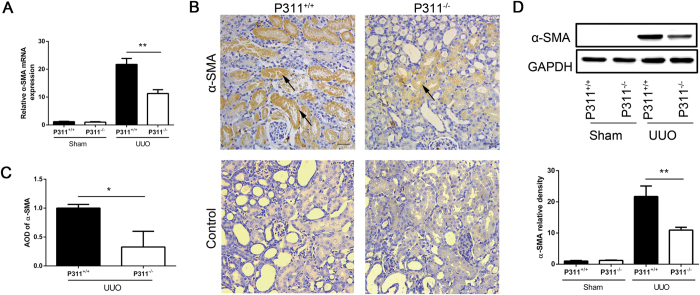
P311 deficiency suppresses α-SMA expression in UUO mice. (**A**) RNA was isolated in kidneys from P311^+/+^ (n = 6) and P311^−/−^ (n = 6) mice after UUO or sham operation. α-SMA mRNA expression was determined by real-time PCR. (**B**) Representative photomicrographs of α-SMA-specific immunohistochemical staining of the obstructed kidneys from both P311^+/+^ and P311^−/−^ mice on day 7. Black arrowhead indicates the α-SMA-positive region. Bottom panel: negative controls for the immunohistochemical staining of α-SMA on the obstructed kidneys from both P311^+/+^ and P311^−/−^ mice. (**C**) α-SMA-positive region was quantified in stained sections from P311^+/+^ (n = 6) and P311^−/−^ (n = 6) mice 7 days after UUO. (D) Western blot analysis of α-SMA protein levels. α-SMA protein levels in each treatment group (n = 3 per group) were quantified. A-D are representative of at least three similar experiments. Scale bar: 100μm. Data are presented as the mean ± SD. *P < 0.05; **P < 0.01.

**Figure 5:**
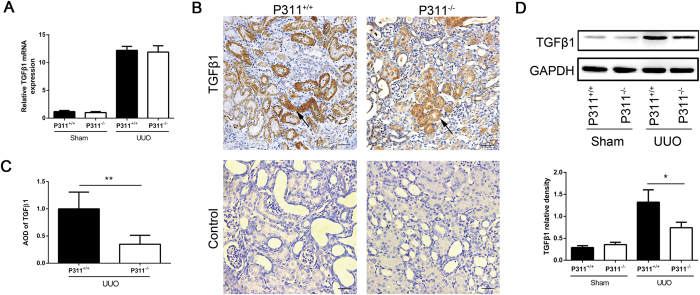
P311 deficiency suppresses TGF-β1 expression in UUO mice. (A) RNA was isolated in kidneys from P311^+/+^ (n = 6) and P311^−/−^ (n = 6) mice 7 days after UUO or sham operation. TGF-β1 mRNA expression was determined by real-time PCR. (B) Representative photomicrographs of TGF-β1-specific immunohistochemical staining of UUO kidneys from P311^+/+^ and P311^−/−^mice on day 7. Black arrowhead indicates the TGF-β1-positive region. Bottom panel: negative controls for the immunohistochemical staining of TGF-β1 on the obstructed kidneys from both P311^+/+^ and P311^−/−^ mice. (**C**) TGF-β1-positive region was quantified in stained sections from P311^+/+^ (n = 6) and P311^−/−^ (n = 6) mice 7 days after UUO. (**D**) Western blot analysis of TGF-β1 protein levels. TGF-β1 protein levels in each treatment group (n = 3 per group) were quantified. A-D are representative of at least three similar experiments. Scale bar: 100μm. Data are presented as the mean ± SD. *P < 0.05; **P < 0.01.

α-SMA is a key marker of myofibroblasts, which are be the interstitial cells that are responsible for fibrosis^25^. Therefore, we examined whether P311 induced α-SMA expression. We assessed α-SMA mRNA and protein levels in both P311^+/+^ and P311^−/−^ kidneys after UUO or sham operation. As shown in Fig. 5A, α-SMA mRNA expression was low in both the P311^+/+^ and P311^−/−^ Sham groups, but was induced by UUO. However, α-SMA mRNA expression was higher in obstructed kidneys from P311^+/+^ mice compared to P311^−/−^ mice (Fig. 4A, 1.93-fold difference, P < 0.001). Immunohistochemical analyses revealed that α-SMA protein localized to the cytoplasm of some tubular epithelial cells and interstitial cells in obstructed kidneys from both P311^+/+^ and P311^−/−^ mice (Fig. 4B). However, the intensity of α-SMA expression, as determined by morphometric quantification, was significantly higher in obstructed kidneys from P311^+/+^ mice compared to P311^−/−^ mice (Fig. 4C, 3.05-fold difference, P = 0.014). Consistent with these observations, western blot analysis showed that α-SMA protein expression was significantly higher in obstructed kidneys from P311^+/+^ mice compared to P311^−/−^ mice (Fig. 4D, 1.48-fold difference, P = 0.032). No α-SMA expression was detected in the Sham groups (Fig. 4D). These data implied that P311 is involved in EMT in renal fibrogenesis.

### P311 deficiency down-regulated TGF-β/Smad signaling

TGF-β1 is involved in renal fibrosis and EMT and is produced in large quantities during renal fibrogenesis. Therefore, we examined whether the decreased fibrosis in obstructed kidneys in P311^−/−^ mice was associated with changes in TGF-β1 production. As shown in Fig. 5A, TGF-β1 mRNA expression significantly increased in the obstructed kidneys in the UUO groups compared to the Sham groups. However, there was no difference between P311^+/+^ and P311^−/−^ mice in either the UUO or Sham groups (Fig. 5A). Immunohistochemical staining showed that TGF-β1 protein was expressed in the cytoplasm of some tubular epithelial cells and interstitial cells in obstructed kidneys P311^+/+^ and P311^−/−^ mice (Fig. 5B). TGF-β1 protein levels were significantly higher in obstructed kidneys from P311^+/+^ mice compared to P311^−/−^ mice, as determined by morphometry (Fig. 5C, 2.86-fold difference, P = 0.003). No TGF-β1-positive tubular epithelial cells were found in the P311^+/+^ and P311^−/−^ Sham groups (data not shown). Western blot analysis also showed that TGF-β1 protein expression was significantly higher in obstructed kidneys from P311^+/+^ mice compared to in P311^−/−^ mice (Fig. 5D, 1.99-fold difference, P = 0.001).

**Figure 6:**
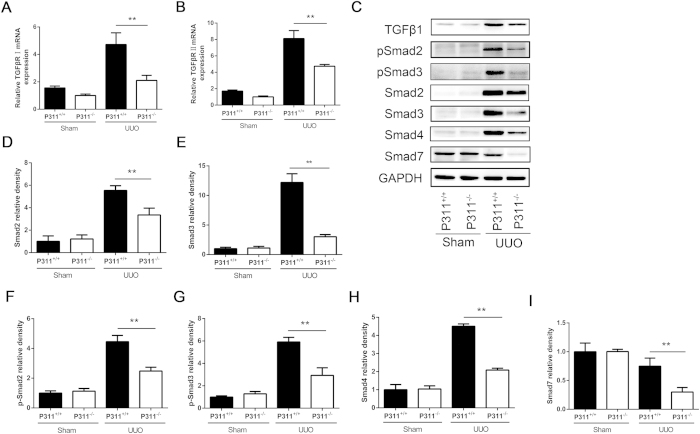
P311 deficiency down-regulates TGF-β/Smad signaling in UUO mice. (**A**) TβR I mRNA expression was determined by real-time PCR in kidneys from P311^+/+^ (n = 6) and P311^−/−^ (n = 6) after mice 7 days after UUO or sham operation. (**B**) TβR II mRNA expression was determined by real-time PCR in kidneys from P311^+/+^ (n = 6) and P311^−/−^ (n = 6) mice 7 days after UUO or sham operation. (**C**) p-Smad2, p-Smad3, Smad2, Smad3, Smad4, Smad7 and GAPDH protein levels were determined in kidneys by western blot. (**D**) Relative density of Smad2 protein (n = 3 per group) in each treatment group. (**E**) Relative density of Smad3 protein (n = 3 per group) in each treatment group. (**F**) Relative density of p-Smad2 protein (n = 3 per group) in each treatment group. (**G**) Relative density of p-Smad3 protein (n = 3 per group) in each treatment group. (**H**) Relative density of Smad4 protein (n = 3 per group) in each treatment group. (**I**) Relative density of Smad7 protein (n = 3 per group) in each treatment group. A-I are representative of at least three similar experiments. Data are presented as the mean ± SD. *P < 0.05; **P < 0.01.

We further examined the type I TGF-β receptor (TβR I), type II TGF-β receptor (TβR II) and downstream targets of Smad signaling. We found that TβR I and TβR II mRNA levels were dramatically increased in both the P311^+/+^ and P311^−/−^ UUO groups compared to the Sham groups but were higher in obstructed kidneys from P311^+/+^ mice compared to P311^−/−^ mice (Fig. 6A, TβR I, P311^+/+^ compared to P311^−/−^, 2.25-fold difference, P = 0.001; Fig. 6B, TβR II, P311^+/+^ compared to P311^−/−^, 1.71-fold difference, P < 0.001). Total Smad2 and Smad3 protein levels were markedly increased in obstructed kidneys from both P311^+/+^ and P311^−/−^ mice, but were significantly higher in the former than in the latter (Fig. 6C,D, Smad2, P311^+/+^ compared to P311^−/−^, 1.65-fold difference, P = 0.007; Fig. 6C,E, Smad3, P311^+/+^ compared to P311^−/−^, 4.03-fold difference, P < 0.001). Moreover, phosphorylated Smad2 (p-Smad2) and phosphorylated Smad3 (p-Smad3) levels were significantly higher in obstructed kidneys from P311^+/+^ mice compared to P311^−/−^ mice (Fig. 6C,F, p-Smad2, P311^+/+^ compared to P311^−/−^, 1.80-fold difference, P = 0.002; Fig. 6C,G, p-Smad3, P311^+/+^ compared to P311^−/−^, 2.01-fold difference, P = 0.001). Smad4 expression was also increased in obstructed kidneys from P311^+/+^ mice compared to P311^−/−^ mice (Fig. 6C,H, 2.16-fold difference, P < 0.001). After UUO, Smad7 protein levels in both P311^+/+^ and P311^−/−^ kidneys decreased compared to the sham operation. Unexpectedly, Smad7 levels were higher in P311^+/+^ kidneys than in P311^−/−^ kidneys after UUO (Fig. 6C,I, 2.50-fold difference, P = 0.008). Taken together, these data showed that P311 deficiency down-regulated TGF-β1 expression, TGF-β1 receptors expression and TGF-β1/Smad signaling activation.

### P311 might be involved in macrophage recruitment in obstructed kidneys

**Figure 7:**
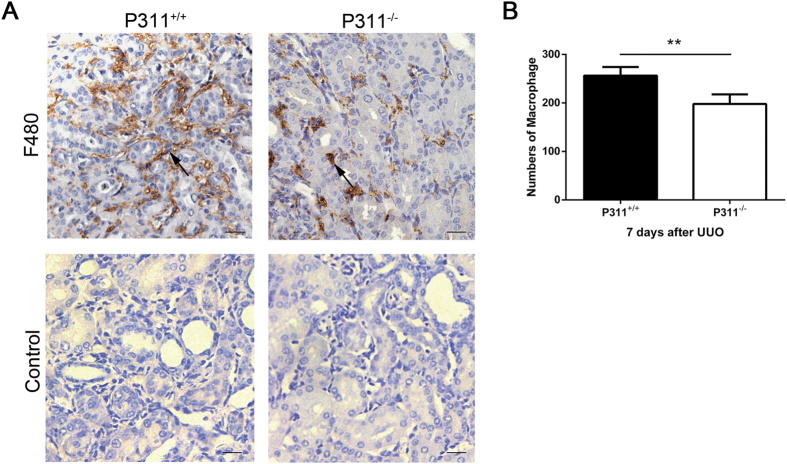
P311 might be involved in macrophage infiltration in UUO mice. (**A**) Representative F480-stained sections of UUO kidneys from P311^+/+^ (n = 6) and P311^−/−^ (n = 6) mice. Black arrowhead indicates the F480-positive cells. Bottom panel: negative controls for the immunohistochemical staining of F480 on the obstructed kidneys from both P311^+/+^ and P311^−/−^ mice. (**B**) The graph represents the total number of F480-positive sections from five contiguous high-powered fields per kidney section. (**A**) and (**B**) are representative of at least three similar experiments. Scale bar: 100 μm. Data are presented as the mean ± SD. *P < 0.05; **P < 0.01.

Fibrosis is the final, common pathological outcome of chronic inflammatory reactions induced by various stimuli, including tissue injury, chronic infections, autoimmune reactions, allergic responses, chemical insults and radiation^26^. Macrophages can synthesize TGF-β to activate fibrogenic cascades^27^. Therefore, we examined whether P311 regulates macrophage infiltration. We used an anti-F480 antibody to detect macrophage infiltration. Immunohistochemical staining revealed few F480-positive cells in the renal interstitium of mice in the Sham groups (data not shown). The number of F480-positive cells were significantly higher in the peritubular areas of obstructed kidneys from P311^+/+^ and P311^−/−^ mice. However, the number of F480-positive cells was much higher in obstructed kidneys from P311^+/+^ mice (256) compared to P311^−/−^ mice (197) (Fig. 7A,B, P = 0.005). Therefore, P311 might be involved in macrophage recruitment to obstructed kidneys.

## Discussion

Renal fibrosis is the final outcome for almost all patients with CKD, for which there are no effective and affordable therapeutic options in the clinical setting, thus creating a heavy socioeconomic burden. Based on the data in this study, we hypothesize that P311 regulates renal fibrogenesis because (1) P311 levels were significantly increased and P311 seem to be co-localized with TGF-β1- and α-SMA-positive regions in renal fibrosis; (2) interstitial collagen deposition, vimentin and α-SMA expression and macrophage infiltration decreased in P311-deficient mice in a mouse model of UUO-induced fibrosis; and (3) P311 deficiency down-regulated tubular TGF-β1 expression, TGF-β1 receptor expression and TGF-β1/Smad signaling activation. This study demonstrate that P311 expression modulates renal fibrogenesis.

Renal fibrogenesis is a dynamic and converging process that involves inflammatory cell infiltration, the activation and expansion of matrix-producing fibroblasts from multiple sources, ECM production and accumulation, tubular atrophy, vascular rarefaction and hypoxia^15^. Here, we confirmed that P311 is highly expressed in the cytoplasm of some tubular epithelial cells in renal fibrosis in both humans and mice, but not in normal human kidney tissue or in sham-operated mouse kidneys. P311 is characterized by the presence of a conserved PEST domain, and the main function of the PEST domain is to mark proteins for rapid degradation by the ubiquitin/proteasome system. In renal fibrosis, the PEST domain might be suppressive to retard P311 degradation. Then to explore the potential role of P311 in renal fibrosis, we used P311^−/−^ mice. These mice have normal kidney organization, histological architecture, blood and urine parameters (Supplementary Figure 1), and P311 is negative in the obstructed kidneys from P311^−/−^ mice (Supplementary Figure 2). However, interstitial collagen deposition and collagen I mRNA levels were down-regulated in obstructed kidneys from P311^−/−^ mice. These results were consistent with our previous observations in human fibroblasts, in which P311 transfection stimulated collagen I expression^10^, and P311 is up-regulated in hypertrophic scar tissue compared to normal skin tissue^24^. These findings suggest that P311 might be involved in renal fibrogenesis. Surprisingly, P311 over-expression in two murine fibroblast cell lines decreased collagen I expression^9^. P311 is central to reactive oxygen species-modulated hepatic stellate cells migration, which may be involved in the development of liver fibrosis. However, P311 knock-down does not affect collagen type I and α-SMA expression^28^. It is difficult to explain this discrepancy with our results, which demonstrated that the different cell type dictates the different functions of P311 where it is expressed. Additional studies are required to clarify this issue.

The tubular epithelium, which undergoes EMT, is an important source of matrix-producing cells and contributes to renal fibrogenesis. During EMT, tubular epithelial cells express vimentin, a fibroblast marker, and α-SMA, a myofibroblast marker^17^. To determine whether P311 regulates renal fibrogenesis via EMT, we examined vimentin and α-SMA expression. We found that vimentin protein levels as well as α-SMA mRNA and protein expression were significantly down-regulated in obstructed kidneys from P311^−/−^ mice. Furthermore, α-SMA-positive regions and acidophilic degeneration seem to be co-localized with P311-positive areas in some tubular epithelial cells. Our previous study also showed that transfecting P311 into human fibroblasts stimulated α-SMA expression^10^. Moreover, P311 transfection into two murine fibroblast cells induced α-SMA expression. P311 and α-SMA co-localize in myofibroblasts during wound healing, and P311 expression occurs upstream of α-SMA expression^9^. These findings support our hypothesis that P311 is involved in renal fibrogenesis EMT.

In renal fibrogenesis, matrix-producing cells integrate various fibrogenic signals and regulate ECM production and assembly. These fibrogenic factors include TGF-β1, platelet-derived growth factor, fibroblast growth factor, connective tissue growth factor and angiotensin II^15^. Among these cytokines, TGF-β1 is the key contributor to EMT in tubular epithelial cells^29^. In the kidneys of patients with IgA nephropathy, P311 protein expression correlates with TGF-β expression and proteinuria^30^. Thus, we next examined whether TGF-β1 was involved in EMT related to P311-mediated renal fibrogenesis. As predicted, obstructed kidneys from P311^−/−^ mice showed impaired TGF-β1 protein expression. Interestingly, there was no difference in TGF-β1 mRNA levels between the P311^−/−^ and P311^+/+^ groups. These results differ from our previous observations in human fibroblasts, which showed that P311 transfection induces TGF-β1 mRNA expression and that interfering with P311 expression in fibroblasts decreases TGF-β1 mRNA expression^10^. The different models (*in vivo* versus *in vitro*) and different cell types (human fibroblasts versus mouse tubular epithelial cells) might be responsible for the discrepancy. P311 binds to latency-associated proteins to regulate TGF-β1 expression^31^. Recently, P311 was reported to bind to eukaryotic translation initiation factor 3 subunit b to promote TGF-β translation and to be the first pan-regulator of TGF-β expression under steady-state conditions^8^,^32^. These findings may explain our results.

The TGF-β/Smad signaling pathway plays a key role in fibrotic disease pathogenesis^33^. TGF-β1 binds to TβR II and then recruits TβR I. The activated complex directly phosphorylates downstream intracellular molecules, including Smad2 and Smad3. Phosphorylated Smad2/3 associates with Smad4 to form a complex that translocates into the nucleus to initiate gene transcription and regulate cell behavior^33^,^34^. TGF-β receptor activation mediates many physiological and pathological processes, including CKD^18^. Increased TβR II expression aggravates ECM accumulation in fibrotic tissue. Inhibition of TGF-β1/Smad2 signaling pathways modulates the development of fibrosis in adriamycin-induced nephropathy^35^. TGF-β/Smad3 signaling promotes renal fibrosis by inhibiting miR-29 and up-regulating miR-21^36^,^37^. Furthermore, inhibiting the endothelial-mesenchymal transition with a Smad3-specific inhibitor (SIS3) delays the early development of diabetic nephropathy^20^. Herein, we found that TβR I and TβR II mRNA expression was induced in the UUO model, but P311 knockout significantly inhibited this up-regulation in this mouse model. Similarly, obstructed kidneys from P311^−/−^ mice showed obviously impaired TGF-β1, p-Smad2, Smad2, p-Smad3, Smad3 and Smad4 protein expression compared to those from P311^+/+^ mice. Taken together, these results indicated that P311 regulates not only TGF-β1 but also the corresponding receptors and the Smad signaling pathway, which may be involved in the regulation of renal fibrosis by P311. However, Meng XM *et al*. found that Smad2 deletion promotes collagen I and III expression in a mouse UUO model^38^. This finding differs from our previous data, and additional studies are required to clarify this discrepancy. TGF-β signaling pathway activation results in the protein expression of inhibitory Smads, such as Smad6 and Smad7. The inhibitor Smad7 can bind to activated TβR I to interfere with TGF-β-induced Smad2/3 activation. In renal fibrosis, Smad7 is renoprotective and blocks the fibrotic effect of TGF-β in renal tubular epithelial cells by inhibiting Smad2 activation and altering TGF-β/Smad3-regulated microRNA expression^39^,^40^. Smad7 over-expression restores the balance of TGF/Smad signaling and exerts a therapeutic effect in CKD^41^. We found that Smad7 protein expression in both P311^+/+^ and P311^−/−^ mice decreased after UUO compared to sham operation, but the decrease was more marked in P311^−/−^ mice. This result was unexpected. Notably, P311 differentially regulated TGF-β1 and Smad proteins. We also found that TGFβ1-positive regions seem to be co-localized with P311-positive regions, α-SMA positive regions and acidophilic degeneration in some tubular epithelial cells within obstructed kidney tissue. We hypothesize that TGF-β/Smad signaling is involved in P311-induced EMT in renal fibrogenesis.

When kidney injury becomes chronic, sustained macrophage infiltration may result the continuous cytokine production, which may become pathological and result in irreversible fibrosis, tissue damage and CKD progression^42^,^43^. Both macrophages and tubular epithelial cells are important sources of UUO-induced changes in renal TGF-β levels, and the interaction between macrophages and tubular epithelial cells plays a role in TGF-β-induced renal fibrosis^44^. Thus, we analyzed macrophage populations and found less macrophage infiltration in obstructed kidneys from P311^−/−^ mice. On the other hand, macrophage-derived TGF-β1 may promote EMT in tubular epithelial cells and activate matrix-producing myofibroblasts^45^. These results suggest that P311 might be involved in macrophage recruitment to increase renal TGF-β levels in obstructed kidneys after UUO.

In summary, our work shows that the P311 gene is involved in renal fibrogenesis, likely by enhancing TGFβ-induced EMT and modulating TGF-β/Smad signaling. Our findings could provide a novel potential target for the control of CKD progression in the future.

## Methods

### Source of human specimens

Renal tissue samples were obtained from the Department of Pathology. This research was approved by the Medical and Ethical Committees of Southwest Hospital, The Third Military Medical University. All human experiments were performed in accordance with the guidelines of The Third Military Medical University.

### Evaluation of renal pathology

Histological analysis was assessed by the experienced pathologists. Biopsies were stained with HE, periodic-Schiff, Masson’s trichrome, and Jones’methenamine silver stain, then scored based on the pathological findings with glomerular sclerosis, tubulointerstitial damage, and vascular sclerosis, which have been previously introduced by Li *et al*.^46^. CKD patients were then classified according to the histologic scores: mildly impaired (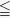
9 points); moderately impaired (10–18 points); severely impaired (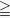
19 points).

### Animals and UUO model preparation

All the animal experimental protocols were approved by the Animal Experimental Ethics Committees of the Third Military Medical University and were performed in accordance with the guidelines of The Third Military Medical University. P311^−/−^ mice were a kind gift from Professor Gregory A Taylor^12^. P311^+/+^ C57BL/6 mice (Charles River Laboratories) were purchased from Beijing VITAL RIVER Company and raised at the Animal Institute of Daping Hospital, The Third Military Medical University. Ten P311^−/−^ mice and ten age-matched P311^+/+^ mice (males; 6–8 weeks old; body weight, 18 to 20 g) were used for the UUO operations. UUO surgeries were performed as previously described^47^,^48^,^49^. Briefly, mice were anesthetized by intraperitoneal injection of 1% sodium pentobarbital (10 μl per gram body weight). In the UUO groups, the left ureter was exposed through a left-flank incision on the back and ligated twice with a 4-0 silk suture. A cut was made between the ligatures to prevent a retrograde urinary tract infection. Sham groups were subjected to a similar procedure without ureteral ligation. After 7 days, the mice were sacrificed. The time point was chosen on the basis of our pre-experiments and the previous study demonstrating that at 7 days, the obstructed kidneys revealed marked tubular dilation and atrophy, interstitial matrix deposition^50^. Subsequently, both kidneys were removed, cut transversely, and either fixed in 4% paraformaldehyde for histopathological studies or snap-frozen in liquid nitrogen for western blot analysis.

### Histological and immunohistochemical analysis

Paraformaldehyde-fixed kidney tissues were embedded in paraffin and sectioned at 4 μm. The sections were dewaxed in xylene, rehydrated in decreasing ethanol concentrations, and stained with HE or Masson’s trichrome for morphological analysis. For immunohistochemical staining, sections were incubated with an antigen retrieval solution at 37 °C for 30 min. After antigen retrieval, sections were incubated with 3% H_2_O_2_ for 20 min at room temperature, with goat serum at 37 °C for 30 min, and with P311 Ab (1:400 dilution; Bioss, China, bs-0427R), α-SMA Ab (1:200 dilution; Abcam, USA, ab5694), TGF-β1 Ab (1:400 dilution; Novus, USA, NB100-91995) or F4/80 Ab (1:100 dilution; Abcam, USA, ab111101) overnight at 4 °C. Sections were washed twice in phosphate buffered saline (PBS) and incubated with a biotinylated secondary antibody for 30 min at 37 °C. Sections were washed, incubated with streptavidin–peroxidase complex for 30 min at 37 °C, and washed once more in PBS. Colorization was monitored using a diaminobenzidine kit (ZSGB-BIO, China, ZLI-9018) and a microscope (OLYMPUS, Japan, CX31). Sections were counterstained with hematoxylin, dehydrated and mounted with resinene. The intensity was quantified in five high-powered fields (×400). In each high-powered field, the total tubular area, including proximal and distal tubules, was first calculated using Image-Pro Plus 6.0 software. TGF-β1- or α-SMA-positive regions were quantified based on the total tubular area. TGF-β1 or α-SMA staining was expressed as the mean ratio of the TGF-β1- or α-SMA-positive area to the total tubular area in the high-powered fields. The number of F4/80-positive cells was counted in five high-powered fields by light microscopy.

### RNA isolation and real-time PCR

RNA was isolated from mouse kidney tissues from the various experimental groups using an RNeasy Mini Kit (QIAGEN, 74104), and cDNA was synthesized using a First Strand cDNA Synthesis Kit (TOYOBO, FSK-100). Real-time PCR analysis of mouse cDNA was performed with the 7500 Real-Time RCR System (Applied Biosystems) and SYBR Green. Expression values were normalized to GAPDH expression. The primer sequences were as follows: TGFβ1: 5′-CCGCAACAACGCCATCTATG-3′ and 3′-CTCTGCACGGGACAGCAAT-5′; TβR I: 5′-TCCCAACTACAGGACCTTTTTCA-5′; and 3′-G

CAGTGGTAAACCTGATCCAGA-5′; TβR II: 5′-ATGGAAGAGTGCAACGATTA

CAT-3 and 3′-TGGCGCAGTTGTCACTGAAAT-5′; Collagen I: 5′-TTCTCCT

GGCAAAGACGGACTCAA-3′ and 3′-AGGAAGCTGAAGTCATAACCGCCA-5′; α-SMA: 5′-CGTACAACTGGTATTGTGCTGGAC-3′ and 3′- TGATGTCACGGACAATCTCACGCT-5′; and GAPDH: 5′-CGTGCCGCCTGGAGAAAC-3′ and 3′-AGTGGGAGTTGCTGTTGAAGTC-5′.

### Western Blot Analysis

A total of 20 μg of kidney lysate was loaded into gels, separated by SDS-PAGE and transferred onto nitrocellulose membranes for immunoblotting analysis. The membranes were blocked with TBS containing 0.1% Tween 20 and 5% BSA for 1 hour at room temperature and incubated overnight at 4 °C with antibodies against α-SMA (Abcam, USA, ab5694), TGFβ1 (Novus, USA, NB100-91995), p-Smad2 (Cell Signaling Technology, USA, 138D4), Smad2 (Cell Signaling Technology, USA, D4384), p-Smad3 (Cell Signaling Technology, USA, C25A9), Smad3 (Cell Signaling Technology, USA, C67H9), Smad4 (Cell Signaling Technology, USA, 9515), Smad7 (Epitomics, USA, 3894-1) and GAPDH (KANGCHEN, China, KC-5G4) to detect target protein expression. The membranes were subsequently washed three times with TBS containing 0.1% Tween 20, incubated with horseradish peroxidase-conjugated secondary antibodies at room temperature for 1 hour and washed again. Finally, the Molecular Imager ChemiDoc^TM^ XRS+ Imaging System (BIO-RAD) was used to detect chemiluminescence.

### Statistical analysis

Statistical comparisons were performed with Student’s t–test or repeated measures ANOVA. Data are presented as the mean ± standard deviation (SD). In all cases, P values less than 0.05 were considered statistically significant.

## Additional Information

**How to cite this article**: Yao, Z. *et al*. P311 promotes renal fibrosis via TGFβ1/Smad signaling. *Sci. Rep*. **5**, 17032; doi: 10.1038/srep17032 (2015).

## Supplementary Material

Supplementary Information

## Figures and Tables

**Table 1:** Baseline clinical data of the patients with chronic kidney disease.

Clinical	With moderate fibrosis	With severe fibrosis	*P*value
	Chronic nephritis	Chronic nephritis	
diagnosis	(n = 10)	(n = 12)	
Sex, M:F	4:6	7:5	\
Age, yr	32.5 ± 11.4	37.5 ± 12.9	NS
Proteinuria	1.67 ± 1.05	2.21 ± 1.46	NS
(g/24h)			
Hb (g/L)	126.90 ± 16.80	93.58 ± 30.53	**
SCr (umol/L)	97.61 ± 30.07	214.50 ± 128.40	*
Plasma albumin	35.02 ± 4.96	31.075 ± 4.13	NS
Pathological	IgA nephropathy (n = 3)	IgA nephropathy (n = 2)	\
diagnosis	FSGS (n = 6)	FSGS (n = 7)	
	DN (n = 1)	DN (n = 2)	
		LN (n = 1)	

Data are presented as the mean ± SD. *P < 0.05; **P < 0.01. NS: not significant; Hb: hemoglobin; SCr: serum creatinine; M: male; F: female; FSGS: focal segmental glomerular sclerosis; DN: diabetic nephropathy; LN: lupus nephritis. Pathological diagnosis were confirmed by kidney biopsy. Ten had moderate fibrosis and the other twelve had severe fibrosis. Analysis of renal fibrosis was determined in 4 μm paraffin-embedded sections stained by HE or Masson’s trichrome.
