# Supplementary material for: P311 promotes renal fibrosis via TGFβ1/Smad signaling
Source: Sci Rep. 2015 Nov 30;5:17032. doi: 10.1038/srep17032 (PMC4663757; doi:10.1038/srep17032)
Supplement: Supplementary Information [file srep17032-s1.pdf]

P311 promotes renal fibrosis via TGFβ1/Smad signaling

Zhihui Yao[1,2], Sisi Yang[1,2], Weifeng He[1,2], Lian Li[1,3], Rui Xu[1], Xiaorong Zhang[1], Haisheng Li[1], Rixing Zhan[1], Wei Sun[1], Jianglin Tan[1], Junyi Zhou[1], Gaoxing Luo[1,2*], Jun Wu[1,2*]

[1]Institute of Burn Research, State Key Laboratory of Trauma, Burn and Combined Injury, Southwest Hospital, The Third Military Medical University, Chongqing 400038, China.

[2]Chongqing Key Laboratory for Disease Proteomics, Chongqing 400038, China.

[3]Department of Nephrology, Southwest Hospital, The Third Military Medical University, Chongqing 400038, China.

*Corresponding author

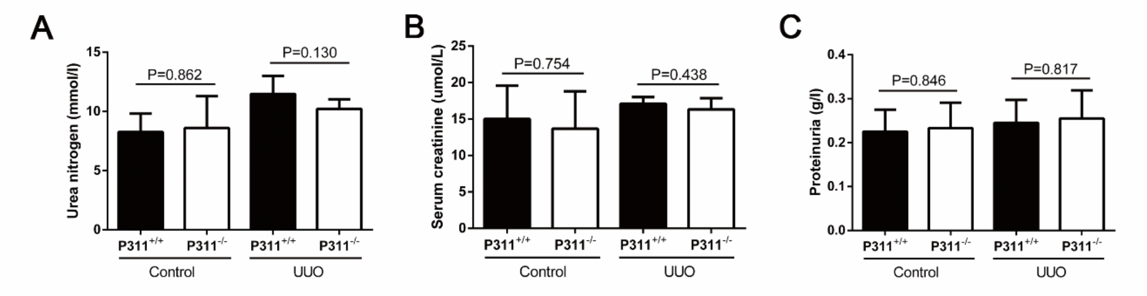

Supplementary figure 1

Baseline blood and urine data of P311$^{+/+}$ and P311$^{-/-}$ mice. (A) Urea nitrogen in blood serum of P311$^{+/+}$ and P311$^{-/-}$ mice (n=4 per group), treated or not treated with unilateral ureteral obstruction (UUO) was analyzed. (B) Serum creatinine in blood serum of P311$^{+/+}$ and P311$^{-/-}$ mice (n=4 per group), treated or not treated with UUO was analyzed. (C) Proteinuria in urine of P311$^{+/+}$ and P311$^{-/-}$ mice (n=4 per group), treated or not treated with UUO was analyzed. Data are presented as the mean ± SD. *P<0.05; **P<0.01.

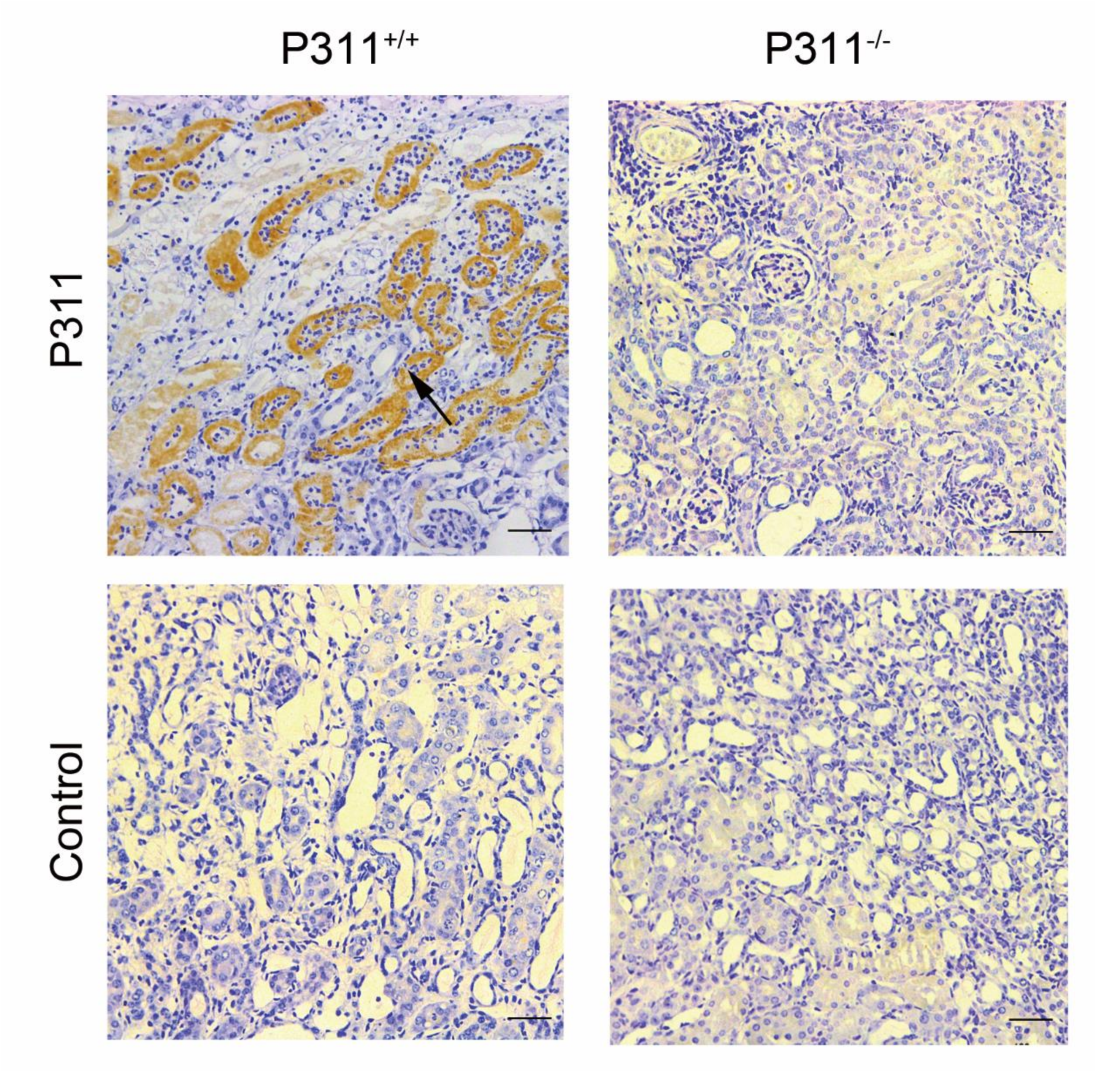

Supplementary figure 2

P311 expression and distribution in kidney tissues from P311$^{+/+}$ (n=6) and P311$^{-/-}$ (n=6) mice, treated with unilateral ureteral obstruction for 7 days in adult mice, as evidenced by immunohistochemistry. Black arrow indicates the P311-positive region. Bottom panel: negative controls for the immunohistochemical staining of P311 on mouse renal fibrosis samples. Scale bar: 100μm.
